# Effects of Salmon Calcitonin on the Concentrations of Monoamines in Periaqueductal Gray in Formalin Test

**DOI:** 10.4274/balkanmedj.galenos.2019.2018.12.88

**Published:** 2019-08-22

**Authors:** Kaveh Rahimi, Javad Sajedianfard, Ali Akbar Owji

**Affiliations:** 1 Department of Basic Sciences, School of Veterinary Medicine, Shiraz University, Shiraz, Iran; 2 Department of Biochemistry, School of Medicine, Shiraz University of Medical Sciences, Shiraz, Iran

**Keywords:** Chromatography, formalin test, high-performance liquid, microdialysis, monoamine oxidase, salmon calcitonin

## Abstract

**Background:**

The receptors of salmon calcitonin, located on certain areas of the brain such as the periaqueductal gray matter, are responsible for pain modulation.

**Aims:**

The effects of intracerebroventricular injection of salmon calcitonin on the behavioral response to pain and on the levels of monoamines in the periaqueductal gray were explored using a biphasic animal model of pain.

**Study Design:**

Animal experiment.

**Methods:**

A total of 45 male rats were divided into four groups (n=6). Salmon calcitonin was injected into the lateral ventricle of the brain (1.5 nmol, with a volume of 5 μL). After 20 min, 2.5% formalin was subcutaneously injected into the right leg claw, and pain behavior was recorded on a numerical basis. At the time of the formalin test, the periaqueductal gray area was microdialized. High-performance liquid chromatography method was used to gauge the levels of monoamines and their metabolites.

**Results:**

Intracerebroventricular injections of salmon calcitonin resulted in pain reduction in the formalin test (p<0.05). The dialysate concentrations of serotonin, dopamine, norepinephrine, 5-hydroxyindoleacetic acid, 3,4-dihydroxyphenylacetic, and 4-hydroxy-3-methoxyphenylglycol increased in the periaqueductal gray area in different phases of the formalin pain test (p<0.05).

**Conclusion:**

Salmon calcitonin reduced pain by increasing the concentrations of monoamines and the metabolites derived from them in the periaqueductal gray area.

The periaqueductal gray (PAG) area has a key role in descending pain control pathways in the brain stem (1). Downstream signals from the nuclei in the brain stem to the spinal cord constrain the dispatch of pain signals through the spinal cord (2). Monoamines play an important role in antinociceptive systems and have a close proximity to one another in the anesthetic centers of the brain (3). Different regions of the brain receive monoaminergic neurons from the PAG area (4).

The c-cells of the thyroid gland synthesize calcitonin (5,6). Calcitonin may be secreted by some cells in the brain (7). CTRa and CTRb are two types of calcitonin receptors whose ability to connect to calcitonin is not very different from each other (8,9). Calcitonin has different species. Salmon calcitonin (sCT) receptors in rats have been distributed with a high density in certain brain areas such as the PAG (10,11,12,13). The intracerebroventricular injection of calcitonin leads to hypocalcemia, which implies that the nervous system controls the blood calcium (14). sCT, which is a nonopioid peptide, has a particular antinociceptive effect. Injecting calcitonin into the middle part of the PAG in the brain of rats reduce acute pain in the thermal withdrawal test (15). The intracerebroventricular injection of sCT causes analgesic effects in tail-flick and hot-plate tests (16). When sCT is injected into the brain of mice, the pain caused by the formalin test is reduced (17). Calcitonin provides an interesting analgesic effect in a series of painful conditions. However, the mechanism of its performance is not well-known. In the current research, the objective is to explore the impact of intracerebroventricular injection of sCT on the level changes of monoamines and the behavioral responses of pain in the PAG area in the formalin pain test.

## MATERIALS AND METHODS

### Animals

In the current research, 45 male Sprague–Dawley rats (250-300 g) were studied. The rats had free access to water and food and were preserved at the temperature of 23±2 °C and in a 12-h light/dark cycle. The protocol of the present study was authorized by the Ethics Committee of the School of Veterinary Medicine of Shiraz University, Shiraz, Iran.

In this study, four groups (n=6) were considered. In the first group, serum physiology was injected intracerebroventricularly (with a volume of 5 mL) and subcutaneously injected in the right leg claw (with a volume of 50 mL). In the second group, serum physiology was injected intracerebroventricularly (with a volume of 5 mL), and then formalin (2.5%) was subcutaneously injected in the right leg claw (with a volume of 50 mL). In the third group, 1.5 nmol of sCT was injected intracerebroventricularly (with a volume of 5 mL), and serum physiology was subcutaneously injected in the right leg claw (with a volume of 50 mL). In the fourth group, 1.5 nmol of sCT (Sigma–Aldrich, USA) (dissolved in serum physiology) was injected intracerebroventricularly (with a volume of 5 mL), and formalin (2.5%) was subcutaneously injected in the right leg claw (with a volume of 50 mL).

### Stereotaxic surgery and microdialysis probe implantation

The rats were anesthetized by injecting pentobarbital sodium peritoneally (50 mg/kg). The guide cannula (with the coordinates: anteroposterior, -0.8; laterality, +1.5; dorsoventral, -3.5) was implanted in the lateral ventricle of the brain. Brain microdialysis probes, with an appropriate length for the PAG area, were constructed based on the Paxinos Atlas. The microdialysis probes were implanted into the PAG area (with the coordinates: anteroposterior, -7.6; laterality, 0.6; dorsoventral, -5.8) (18).

### Pain assessment and the microdialysis of the PAG area

The formalin test is applied for the assessment of pain sensation. In this test, if the rat does not exhibit any unusual behavior, then it is given the score 0; if the rat’s leg claw is on the floor of the chamber and it does not put its weight on the leg claw, then it is given the score 1; if the rat strikes the floor of the chamber with a leg claw or pulls up its legs to the abdominal area, then it is given the score 2; and lastly, if the rat bites or licks the injection location, then it is given the score 3 (19). The subcutaneous injection of formalin in the leg claw causes a two-phase pain response in the rat. The first 5 minutes (min) after formalin injection comprise the first phase. The second phase begins from the end of the fifteenth min to the end of the sixtieth min. In addition, the interphase starts from the end of the fifth min to the end of the fifteenth min (20).

After 24 h, the rats were put in a microdialysis chamber and were permitted to adjust to the environment for 15 min. A Hamilton syringe (10 µL) was used for intracerebroventricular injections of sCT. The pain test was performed 20 min after the injection of sCT. The PAG area was perfused with artificial cerebrospinal fluid with a flow rate of 2.0 µL/min using a syringe pump (WPI, serum physiology 210). The dialysis samples were gathered at 15 min intervals [(S1), the base sample; (S2), base sample with sCT or serum physiology effects; (S3-S6), the four samples corresponding to the various times of the formalin test with sCT or serum physiology effects; and (S7, S8), the two samples collected after finishing the pain test]. The artificial cerebrospinal fluid used was a combination of (in mM) CaCl_2_ (1), KCl (3), NaH_2_PO_4_ (1.25), NaCl (114), NaHCO_3_ (26), MgSO_4_ (2), NaOH (1), and glucose (10) with pH of 7.40.

### Chemical assays

High-performance liquid chromatography-electrochemical detection method was utilized to determine the concentrations of dopamine, serotonin, norepinephrine, and their metabolites in the dialysis of the PAG area (n=6). After preparing and adding the internal standard (14.3 µL), the samples were placed into an high-performance liquid chromatography column (Eurospher reverse-phase column, 100-5 C18, 250×4.6 mm), a pump (Knauer), and an electrochemical detector (Amperometric Detector EC 3000). The oxidizing potential of the working electrode was set at +750 mV versus the Ag/Cl reference electrode. The mobile phase comprised a combination of ethylenediaminetetraacetic acid (30 mg), sodium phosphate (8.4 g), 1-octane-sulfonic acid (360 mg), and 16% methanol (in 1000 mL of water with high-performance liquid chromatography grade, pH=4.5). The mobile phase had a flow rate of 1.0 mL/min.

### Histological substantiation

The rats were put down using a high dose of diethyl ether (MERK, Germany) after completing each experiment. After 72 h, the rats’ brains were placed in 10% formalin. The positions of the guide cannula in the ventricle and the probes of microdialysis in the PAG area of all the brains were verified according to the Paxinos Atlas (18).

### Statistical analysis

The SPSS software (version 16) was used to analyze the data. According to the distribution and homogeneity of variances, a normalization test was performed on the data (SPSS Kolmogorov–Smirnov test). Since the data were normal, one-way ANOVA was used to evaluate the groups. Duncan’s tests were performed as post-hoc analysis. P˂0.05 was taken as the significance level.

## RESULTS

### Pain assessment

The results of the pain behavior responses at various phases of the formalin pain test are shown in Table 1.

**Figure 1:**
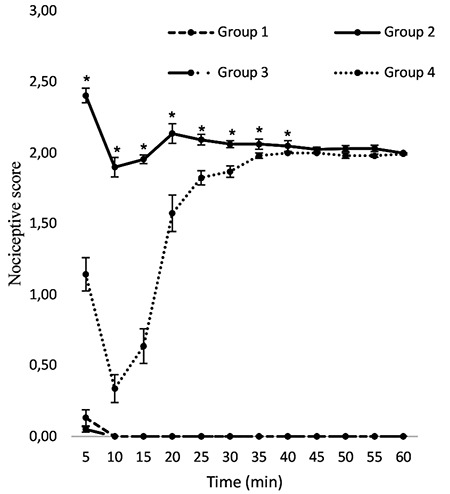
The nociceptive score in different groups. [Group 1: Normal saline was injected intracerebroventricularly, and normal saline was injected subcutaneously in the hind paw; Group 2: normal saline was injected intracerebroventricularly, and 2.5% formalin was injected subcutaneously; Group 3: Salmon calcitonin with a dose of 1.5 nmol was injected intracerebroventricularly, and normal saline was injected subcutaneously; Group 4: Salmon calcitonin with a dose of 1.5 nmol was injected intracerebroventricularly, and 2.5% formalin was injected subcutaneously]; *Significant differences between groups 2 and 4 (p<0.05) (mean ± standard deviation)

A significant decrease in the nociceptive behavioral score in the first phase of the formalin pain test was observed in the fourth group (1.46±0.08) when sCT was injected into the ventricle of the brain as compared to that of the second group (2.4±0.05) (Figure 1). A significant decrease in the nociceptive behavioral score when sCT was injected in interphase of the formalin pain test was observed in the fourth group (0.35±0.06) as compared to that of the second group (1.92±0.03) (Figure 1). Furthermore, administering sCT through injection in the chronic phases of the formalin pain test of the fourth group (1.87±0.03) significantly reduced nociception after formalin injection compared to that of the second group (2.05±0.01) (Figure 1).

### The concentrations of monoamines in the PAG area

The third dialysis samples (S3), which included the artificial cerebrospinal fluid gathered from the PAG area in 0-15 min time intervals after formalin injection, correlated to the first phase and interphase of the formalin pain test. The fourth, fifth, and sixth dialysis samples (S4-S6) obtained within the time intervals of 15-30, 30-45, and 45-60 min, respectively, after formalin injection correlated to the second phase of the formalin pain test.

### *Serotonin*

**Figure 2:**
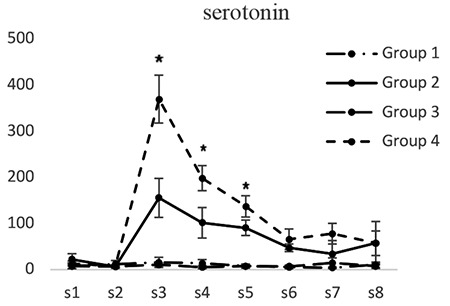
Concentrations of serotonin in different groups. [S1 to S8 represent the collected samples of dialysis from the periaqueductal gray at different times. Base sample without medication effect (S1), base sample with medication effect (S2), four samples related to different times of the formalin test (S3-S6), and two samples after completion of formalin test (S7, S8)]; *Significant differences between groups 2 and 4 (p<0.05) (mean ± standard deviation)

Serotonin concentrations in groups 1 and 3 did not have significant differences at any of the tested times. The serotonin concentration (pg/mL) in dialysis sample 3 in the second group (155.42±31.12) was significantly lower than that of the fourth group (369.11±51.92) (p<0.05). Serotonin concentrations in dialysis samples 4 and 5 in the second group (101.33±11.36 and 90.16±17.61) were significantly lower than those of the fourth group (198.27±17.06 and 136.23±19.32) (p<0.05). Serotonin concentration in dialysis sample 6 in the second group was lower than that of the fourth group. However, the difference was not significant (Figure 2).

### *5-Hydroxyindoleacetic acid*

**Figure 3:**
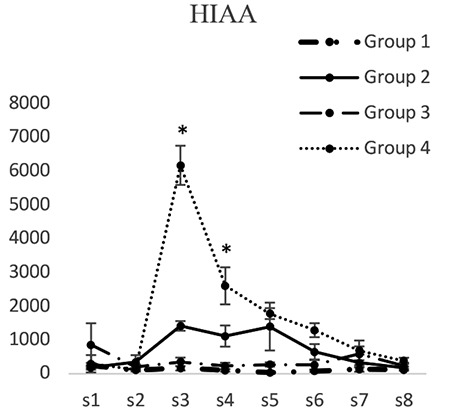
Concentrations of 5-hydroxyindoleacetic acid in different groups. [S1 to S8 represent the collected samples of dialysis from the periaqueductal gray at different times. Base sample without medication effect (S1), base sample with medication effect (S2), four samples related to different times of the formalin test (S3-S6), and two samples after completion of formalin test (S7, S8)]; *Significant differences between groups 2 and 4 (p<0.05) (mean ± standard deviation)

5-Hydroxyindoleacetic acid concentrations did not have significant differences in groups 1 and 3 at any of the time intervals. 5-Hydroxyindoleacetic acid concentration (pg/mL) in dialysis sample 3 in the second group (1416.142±79.74) was significantly less than that of the fourth group (6166.580±16.28) (p<0.05). 5-Hydroxyindoleacetic acid concentration in dialysis sample 4 in the second group (1115.312±66.18) was significantly less than that of the fourth group (2061.548±40.01) (p<0.05). 5-Hydroxyindoleacetic acid concentrations in dialysis samples 5 and 6 in the fourth group did not show any significant differences compared to those of the second group (Figure 3).

### *Dopamine*

**Figure 4:**
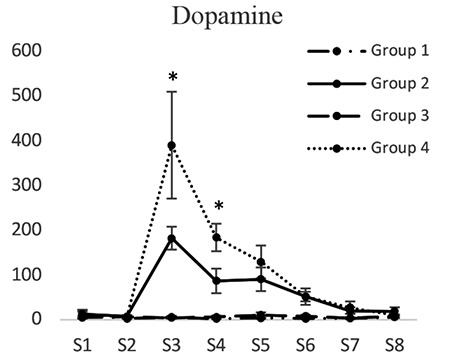
Concentrations of dopamine in different groups. [S1 to S8 represent the collected samples of dialysis from the periaqueductal gray at different times. Base sample without medication effect (S1), base sample with medication effect (S2), four samples related to different times of the formalin test (S3-S6), and two samples after completion of formalin test (S7, S8)]; *Significant differences between groups 2 and 4 (p<0.05) (mean ± standard deviation)

Dopamine concentrations in groups 1 and 3 did not have significant differences at any of the tested times. Dopamine concentration (pg/mL) in dialysis sample 3 in the second group (182.25±32.58) was significantly lower than that of the fourth group (390.119±12.29) (p<0.05). Dopamine concentration in dialysis sample 4 in the second group (88.27±18.42) was lower than that of the fourth group (183.30±19.68) (p<0.05). Dopamine concentrations in dialysis samples 5 and 6 in the second group were lower than those of the fourth group. Nevertheless, the difference was not significant (Figure 4).

### *3,4-Dihydroxyphenylacetic*

**Figure 5:**
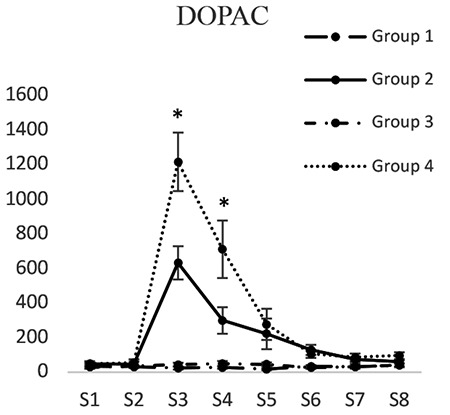
Concentrations of 3,4-dihydroxyphenylacetic in different groups. [S1 to S8 represent the collected samples of dialysis from the periaqueductal gray at different times. Base sample without medication effect (S1), base sample with medication effect (S2), four samples related to different times of the formalin test (S3-S6), and two samples after completion of formalin test (S7, S8)]; *Significant differences between groups 2 and 4 (p<0.05) (mean ± standard deviation)

3,4-Dihydroxyphenylacetic concentrations in groups 1 and 3 did not have significant differences at any of the time intervals. 3,4-Dihydroxyphenylacetic concentration (pg/mL) in dialysis sample 3 in the second group (630.97±97.02) was significantly less than that of the fourth group (1214.169±29.01) (p<0.05). Dopamine concentration in dialysis sample 4 in the second group (298.76±38.34) was less than that of the fourth group (709.166±40.25) (p<0.05). 3,4-Dihydroxyphenylacetic concentrations in dialysis samples 5 and 6 in the fourth group did not show any significant differences compared to those of the second group (Figure 5).

### *Norepinephrine*

**Figure 6:**
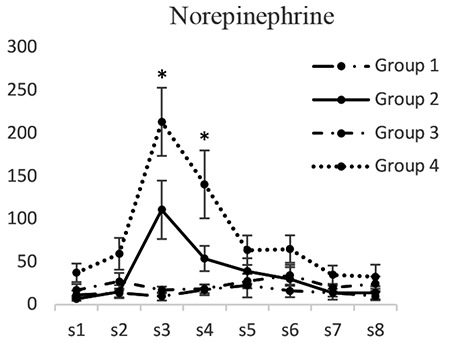
Concentrations of norepinephrine in different groups. [S1 to S8 represent the collected samples of dialysis from the periaqueductal gray at different times. Base sample without medication effect (S1), base sample with medication effect (S2), four samples related to different times of the formalin test (S3-S6), and two samples after completion of formalin test (S7, S8)]; *Significant differences between groups 2 and 4 (p<0.05) (mean ± standard deviation)

Norepinephrine concentrations in groups 1 and 3 did not have significant differences at any of the time intervals. Norepinephrine concentration (pg/mL) in dialysis sample 3 in the second group (110.34±19.13) was significantly lower than that of the fourth group (213.39±17.85) (p<0.05). Norepinephrine concentration in dialysis sample 4 in the second group (53.14±19.79) was lower than that of the fourth group (140.39±25.61) (p<0.05) (Figure 6).

### *4-Hydroxy-3-methoxyphenylglycol*

**Figure 7:**
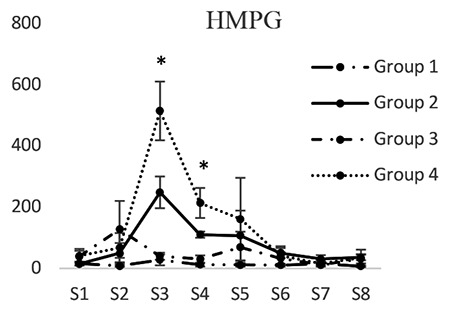
Concentrations of 4-hydroxy-3-methoxyphenylglycol in different groups. [S1 to S8 represent the collected samples of dialysis from the periaqueductal gray at different times. Base sample without medication effect (S1), base sample with medication effect (S2), four samples related to different times of the formalin test (S3-S6), and two samples after completion of formalin test (S7, S8)]; *Significant differences between groups 2 and 4 (p<0.05) (mean ± standard deviation)

4-Hydroxy-3-methoxyphenylglycol concentrations in groups 1 and 3 did not have significant differences at any of the time intervals. 4-Hydroxy-3-methoxyphenylglycol concentration (pg/mL) in dialysis sample 3 in the second group (248.51±41.78) was significantly less than that of the fourth group (514.95±42.89) (p<0.05). 4-Hydroxy-3-methoxyphenylglycol concentration in dialysis sample 4 in the second group (110.13±10.05) was less than that of the fourth group (213.49±16.09) (p<0.05). 4-Hydroxy-3-methoxyphenylglycol concentrations in dialysis samples 5 and 6 in the second group were less than those of the fourth group. However, the difference was not significant (Figure 7).

## DISCUSSION

The nociceptive behavioral scores in the first, inter, and second phases of the formalin pain test were significantly reduced by the introduction of sCT through intracerebroventricular injection. sCT increased the concentrations of serotonin, dopamine, norepinephrine, 5-Hydroxyindoleacetic acid, 3,4-Dihydroxyphenylacetic, and 4-Hydroxy-3-methoxyphenylglycol in the first phase and interphase of the formalin pain test in the PAG area. sCT also increased the concentrations of serotonin, dopamine, norepinephrine, and their metabolites at the start of the second phase of the formalin pain test. In our previous study, the other calcitonin family peptide (calcitonin gene-related peptide) showed similar effects. Subsequently, intracerebroventricular injection of calcitonin gene-related peptide reduced pain after the injection of formalin. The dialysis of PAG also showed that the intracerebroventricular injection of calcitonin gene-related peptide increased the concentrations of serotonin, norepinephrine, dopamine, 5-Hydroxyindoleacetic acid, 4-Hydroxy-3-methoxyphenylglycol, and 3,4-Dihydroxyphenylacetic in the PAG in the formalin pain test (21).

sCT has analgesic effects on both somatic, such as muscle and bone pains, and visceral pains, such as migraine headaches. sCT has recently been shown to have the ability to attenuate migraine-like pains by c-fos expression and regulating the release of calcitonin gene-related peptide at different levels (22). eCT, a synthetic derivative of eel calcitonin, exhibits analgesic effects on radicular pain by modulating mRNA-expression of voltage-gated sodium channels. Thus, it can be stated that patients who have radicular pain or need long-term treatments prefer calcitonin for their therapy (23). sCT can inhibit the progression of facet joint syndrome in ovariectomized rats. This potential is ascribed to the inhibitory impacts of sCT on apoptosis, cartilage metabolism imbalance, and bone remodeling (24). PCR analysis shows that the chronic constriction injury causes the upregulation of tetrodotoxin-sensitive Nav.1.3 mRNA. It also leads to the downregulation of mRNA of Nav1.8 and Nav1.9 on the dorsal root ganglion. This will, in turn, enhance the excitability of peripheral nerves. Elcatonin can play an important role in reversing these changes (25).

The results of the current study demonstrated that the intracerebroventricular injection of sCT significantly decreased nociception during the first phase and interphase of the formalin pain test. Candeletti and Ferri also reported similar effects in mice (17). Different mechanisms have been attributed to the analgesic effects of calcitonin. However, no study has shown a change in the concentration of monoamines in the analgesic systems of the brain. Our results indicate that the concentrations of serotonin, dopamine, norepinephrine, and their metabolites have increased in the PAG area in the first phase and interphase of the formalin pain test. Therefore, it seems that the monoaminergic pathways in the brain mediate the analgesic effects observed in the first phase and interphase of the formalin pain test.

The central serotonergic system might have a role in the analgesic effect caused by calcitonin (26). The intracerebroventricular injection of calcitonin results to the increase in serotonin contents in several regions of the brain (27). Serotonin has an analgesic effect on chronic pain in humans and animals (28). Calcitonin in ovariectomized rats can cause the release of glutamate from C-afferent fibers and can also normalize the expression of sodium channel in damaged peripheral nerves (29). Immunohistochemical studies have also shown that the PAG area that contains dopaminergic neurons is involved in the analgesic system. The injection of the dopamine antagonist in the vPAG reduces the analgesic effects of heroin and morphine (30). In addition, norepinephrine and 4-Hydroxy-3-methoxyphenylglycol concentrations are increased in the locus coeruleus in the formalin test (31). locus coeruleus has projections to different areas of the brain such as the PAG (32).

In the second phase of the formalin pain test, pain-related behaviors were significantly decreased with the intracerebroventricular injection of sCT. The concentrations of serotonin and 5-Hydroxyindoleacetic acid increased at 15-45 min and 15-30 min time intervals after formalin injection, respectively. The serotonergic system in the PAG area plays a key role in pain modulation. Administering serotonin agonist in the PAG area reduces pain (33). Dopamine and its metabolite (3,4-Dihydroxyphenylacetic) concentrations increased at 15-30 min time interval in the second phase of the formalin pain test. It is noteworthy that a network of dopaminergic neurons is distributed throughout the mesencephalon (34). sCT also increased the concentrations of norepinephrine and its metabolite at 15-30 min time interval after the injection of formalin. Administering norepinephrine in the dorsal part of the PAG area showed that norepinephrine plays a role in pain perception (34).

sCT also has clinical applications. Since the 1970s, sCT has been applied as a nasal spray or injection to treat osteoporosis and other metabolic bone diseases (35). sCT has also been used for the treatment of postmenopausal osteoporosis. Although the nasal application is less effective than the injectable formulation, it has been more frequently used. sCT increases bone mineral density (36,37). Nasal calcitonin spray has been more effective than gabapentin in treating patients with lumbar spinal stenosis (38). Moreover, nasal sCT has been recommended for ameliorating acute osteoporotic distal radius fractures (39). A study focused on a novel oral sCT (SMC021) found that this sCT failed to meet its primary objective, which is to reduce vertebral fractures in postmenopausal women suffering from osteoporosis. The study suggested that more researches should be done on the delivery of peptides (40). Despite its analgesic effects, oral sCT does not have renewable benefits for patients suffering from knee osteoarthritis (41). Studies suggest that calcitonin can alleviate back pains of patients suffering from neuropathic pain or osteoporosis by altering the expression of channels or receptors (29,42).

In the current study, the intracerebroventricular injection of sCT reduced pain in the formalin pain test. The observed effect may be due to an increase in serotonin, dopamine, norepinephrine, and their metabolites in the PAG area or the related nuclei.

## Figures and Tables

**Table 1:**
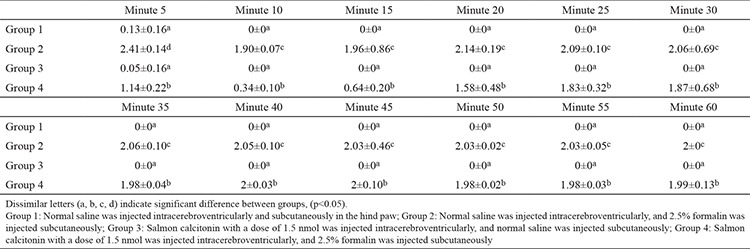
Pain-related behaviors (mean ± standard deviation) in formalin test
